# Stimulation of Epithelial Sodium Channels in Endothelial Cells by Bone Morphogenetic Protein-4 Contributes to Salt-Sensitive Hypertension in Rats

**DOI:** 10.1155/2020/3921897

**Published:** 2020-10-28

**Authors:** Xu Yang, Na Niu, Chen Liang, Ming-Ming Wu, Liang-Liang Tang, Qiu-Shi Wang, Jie Lou, Bin-Lin Song, Wei-Wan Zheng, He-Ping Ma, Zhi-Ren Zhang

**Affiliations:** ^1^Departments of Clinical Pharmacy and Cardiology, Harbin Medical University Cancer Hospital, Institute of Metabolic Disease, Heilongjiang Academy of Medical Science, Key Laboratories of Education Ministry for Myocardial Ischemia Mechanism and Treatment, Harbin 150081, China; ^2^Department of Pharmacy, The 1st Affiliated Hospital of Harbin Medical University, Harbin 150001, China; ^3^Department of Physiology, Emory University School of Medicine, Atlanta, Georgia 30322, USA

## Abstract

Previous studies have shown that high salt induces artery stiffness by causing endothelial dysfunction via increased sodium influx. We used our unique split-open artery technique combined with protein biochemistry and *in vitro* measurement of vascular tone to test a hypothesis that bone morphogenetic protein 4 (BMP4) mediates high salt-induced loss of vascular relaxation by stimulating the epithelial sodium channel (ENaC) in endothelial cells. The data show that high salt intake increased BMP4 both in endothelial cells and in the serum and that exogenous BMP4 stimulated ENaC in endothelial cells. The data also show that the stimulation is mediated by p38 mitogen-activated protein kinases (p38 MAPK) and serum and glucocorticoid-regulated kinase 1 (Sgk1)/neural precursor cell expressed developmentally downregulated gene 4-2 (Nedd4-2) (Sgk1/Nedd4-2). Furthermore, BMP4 decreased mesenteric artery relaxation in a benzamil-sensitive manner. These results suggest that high salt intake stimulates endothelial cells to express and release BMP4 and that the released BMP4 reduces artery relaxation by stimulating ENaC in endothelial cells. Therefore, stimulation of ENaC in endothelial cells by BMP4 may serve as another pathway to participate in the complex mechanism of salt-sensitive (SS) hypertension.

## 1. Introduction

High dietary sodium chloride intake has been related to hypertension and its target organ damage [1]. It has long been known that a high-salt diet increases the stiffness of endothelial cells [2]. High salt-induced endothelial cell stiffness promotes increased arterial stiffness by elevating transforming growth factor (TGF)-*β* levels [3]. Recent clinical studies have also shown that high salt intake induces large artery stiffness in a salt-sensitive population [4]. Therefore, investigation of the molecular mechanism by which high salt causes endothelial cell dysfunction and artery stiffness has clinical significance for improving the management of salt-sensitive hypertension. Previous studies have shown that elevated sodium influx accounts for endothelial cell stiffness [5]. The pathways for sodium influx were unclear, until we recently recorded the single-channel activity of ENaC in endothelial cells [6]. However, the complex mechanism by which high dietary salt stimulates ENaC in endothelial cells remains to be further determined.

It has been well established that overproduction of TGF-*β*1 contributes to salt-sensitive hypertension [7]. Since TGF-*β*1 stimulates ENaC expression in cultured human cortical collecting duct principal cells [8], high salt may stimulate ENaC in endothelial cells by promoting TGF-*β* production. However, conflicting results also show that TGF-*β*1 inhibits renal and lung ENaC [9–11]. The conflicting results may be due to the different doses of TGF-*β* because TGF-*β* can induce biphasic effects [12]. TGF-*β* consists of a complex superfamily that binds to its also complex receptors [13]. Among the various ligands of TGF-*β* receptors, the multifunctional growth factor BMP4, which belongs to the TGF-*β* superfamily [14], attracted our particular attention. We wanted to test the role of BMP4 in mediating high salt-induced ENaC activity in endothelial cells, because previous studies have shown that administration of BMP4 induces hypertension in mice [15]. Additionally, overexpression of BMP4 may mediate hypertension in patients with colorectal adenocarcinoma, because previous studies have shown that BMP4 is overexpressed in human colorectal adenocarcinoma cells [16, 17] and that hypertension often occurs in patients with adenocarcinoma [18, 19]. These studies suggest that BMP4 may act as a signaling molecule to mediate the development of hypertension.

In this study, we aimed to characterize the role and the potential mechanisms of BMP4-mediated ENaC channels in the regulation of blood pressure in salt-sensitive hypertension rats. We performed cell-attached patch-clamp, perforated whole-cell patch-clamp, isometric myography, and Western blotting experiments to address this question in salt-sensitive rats, using a preparation of the freshly isolated vascular endothelium. We hypothesized that excessive activation of ENaC by BMP4 is a central contributor to endothelial injury disease and salt-sensitive hypertension.

## 2. Materials and Methods

### 2.1. Animals

All animal care and experimental procedures were approved by the Harbin Medical University Animal Supervision Committee. All studies involving animals are reported in accordance with the ARRIVE guidelines for reporting experiments involving animals [20, 21].

Male Sprague-Dawley (SD) rats and male Dahl salt-sensitive (SS) rats weighing 220-240 g (8 weeks) were used in the present study. The age of the animals in the various groups was matched. SD and SS rats were divided randomly into two groups: normal salt (NS) diet (0.3% NaCl, *w*/*w* for three weeks) and high-salt (HS) diet (5% NaCl, *w*/*w* for three weeks). Water and food were supplied *ad libitum*. The blood pressure of awake rats was measured weekly by tail-cuff plethysmography (CODA, 20310, Kent Scientific Corporation, USA). To minimize stress-induced fluctuations in blood pressure, the rats were trained to get used to daily blood pressure measurement for 3 days before the experiments began. Before detection of tail artery pulsations, the rats were placed in the holder for 15 min at 37°C. The higher and lower readings were discarded and the tail arterial blood pressure was obtained from the average of six measurements. Then, blood was collected from the main abdominal artery for serum BMP4 detection using an ELISA kit (LS-F23161, Lifespan Biosciences, USA).

### 2.2. Primary Culture of Rat Mesenteric Artery Endothelial Cells

Mesenteric artery endothelial cell (MAEC) primary culture was carried out using a previously described method [22, 23]. Briefly, SD and SS rats were euthanized by CO_2_ suffocation. When the abdomen was opened, the whole mesenteric vascular bed was dissected out, and all the vein branches of the mesenteric bed were rapidly excised under a dissecting microscope. The remained arterial branched were digested with 0.2 mg/mL collagenase IA (C9891, Sigma-Aldrich, USA), for 50 min at 37°C with mild shaking. When the enzymes were digested, endothelial cells were collected by centrifugation at 1200 rpm for 5 min. The cells were resuspended in DMEM solution (100 U/mL penicillin, 100 *μ*g/mL streptomycin, and 20% fetal bovine serum) and cultured in gelatin-coated Petri dishes. Nonadherent adherent cells were removed after 1 hour, and the adherent endothelial cells were cultured at 37°C with 5% CO_2_ for 3-5 days. These cells were used for experiments or, respectively, treated with BMP4 (20 ng/mL, 314-BP-010, R&D, USA), noggin (100 ng/mL, 6057-NG-025, R&D, USA), SB202190 (10 *μ*M, S7067, Sigma-Aldrich, USA), mannitol (80 mM), and additional NaCl (40 mM) in the DMEM culture medium (10-013-CVRC, Corning, USA) for 24 hours.

### 2.3. Electrophysiology Recording

As previously described, in situ cell-attached patch-clamp recordings of ENaC single-channel currents were performed using intact vascular endothelia [22–24], 3 weeks after feeding NS and HS diet. Mesenteric arteries and physiological saline solution (PSS) were added to a Petri dish for dissection. PSS contained (in mM): 137 NaCl, 5.4 KCl, 0.05 CaCl_2_, 0.4 KH_2_PO_4_, 0.4 Na_2_HPO_4_, 4.4 NaHCO_3_, and 10 HEPES (pH 7.4 with HCl). Second-order branches of mesenteric arteries were dissected and placed on a 5 × 5-mm cover glass coated with L-polylysine, or the primary culture of rat MAECs was transferred to a chamber mounted on an inverted Nikon microscope (Tokyo, Japan), allowing direct access to the endothelial cell layer. Single-channel ENaC currents were recorded in a cell-attached configuration with an Axon Multiclamp 200B amplifier (Axon Instruments; Foster City, CA, USA) at room temperature (22–24°C). Patch pipettes were pulled from borosilicate glass with a Sutter P-97 horizontal puller, and the pipette resistances ranged from 6 to 10 M*Ω* in the bath. The bath and the pipette solutions contained (in mM): 135 NaCl, 4.5 KCl, 1 MgCl_2_, 1 CaCl_2_, 5 HEPES, and 5 Na-HEPES (pH 7.4 with NaOH). After gigaseal formation, the single-channel currents were recorded immediately at least over 30 min. The data were acquired by application of 0 mV to the patch pipettes and then sampled at 5 kHz and low-pass filtered at 1 kHz with Clampex 10.2 software (Molecular Devices, Sunnyvale, CA, USA). Single-channel traces were further filtered at 30 Hz prior to analysis. The open probability (*P*_*O*_) was calculated according to the following formula: *P*_*O*_ = *NP*_*O*_/*N*; the apparent number of active channels in the patch was represented by *N* (*N* was estimated from the current amplitude histogram).

Perforated whole-cell recording was performed in MAECs, using pipettes of 1~2 M*Ω* resistance as previously described [6]. Bath saline was as the same described for single-channel recordings, but with the addition of 10 mM D-glucose. The pipette solution contained (in mM): 40 KCl, 100 K-gluconate, 1 MgCl_2_, 1 CaCl_2_, 0.1 EGTA, 4 Na_2_ATP, 10 glucose, 10 HEPES, and 2 GTP (pH 7.2 with KOH). For perforated patch whole-cell recording, the pipette solution was supplemented with 20 *μ*g/mL amphotericin B (46006-100MG, Fluka, USA). Representative current sweeps evoked by a voltage ramp from 60 mV to -100 mV (holding potential at 40 mV) for a duration of 500 ms. The data were generated by an Axon Multiclamp 200B amplifier (Axon Instruments; Foster City, CA, USA) at room temperature (22–24°C) in MAECs isolated from SS rats. The cells were cultured with or without an additional 20 ng/mL BMP4 in the culture medium for 24 hours, in the presence or absence of 1 *μ*M benzamil.

### 2.4. Wire Myograph Studies

Isolated mesenteric artery ring responsiveness was measured using an isometric myograph (Danish Myo Technology, Aarhus, Denmark), as previously described [22, 23, 25, 26]. Briefly, the isolated second-order mesenteric arteries were carefully cleaned of adherent adipose tissue and cut into 1.8-2 mm lengths. Before evaluating the relaxation effect of ACh and NTG, some rat mesenteric artery rings were incubated for 4 hours in DMEM solution (100 U/mL penicillin, 100 *μ*g/mL streptomycin, and 20% fetal bovine serum) and were placed in a CO_2_ incubator with 95% O_2_ plus 5% CO_2_ with BMP4 (50 ng/mL), noggin (a BMP4 antagonist, 200 ng/mL), or SB202190 (a p38 MAPK inhibitor, 20 *μ*M). Then, changes in the isometric tone of mesenteric arteries were recorded in a wire myograph equilibrated in PSS at 37°C and bubbled with a mixture of 5% CO_2_ in 95% O_2_. The arterial rings were given a resting tension of 3 mN and then allowed to equilibrate for 60 min prior to experiment. The integrity of the functional endothelium was tested by obtaining a relaxation to ACh (1 *μ*M) in rings precontracted with phenylephrine (10 *μ*M). The endothelium was considered intact when such an ACh–induced relaxation was more than 85% of the precontraction value to phenylephrine. After a 60 min stabilization period, KPSS that contained (in mM) 82.4 NaCl, 60 KCl, 0.05 CaCl_2_, 0.4 KH_2_PO_4_, 0.4 Na_2_HPO_4_, 4.4 NaHCO_3_, and 10 HEPES (pH 7.4 with HCl) was added to the chambers and then washed out with PSS until a reproducible maximal contraction was achieved. Endothelium-dependent relaxation (EDR) and endothelium-independent relaxation were measured by testing concentration responses to the cumulative addition of acetylcholine (ACh, 0.1 nM to 100 *μ*M) or nitroglycerin (NTG, 0.1 nM to 10 *μ*M) to precontracted rings with phenylephrine (Phe, 10 *μ*M). Some rings were incubated with benzamil (an ENaC blocker, 1 *μ*M, B2417, Sigma-Aldrich, USA) for 1 hour before assessing their relaxation response to ACh and NTG.

### 2.5. Transfection of Plasmids

A lentivirus (LV) vector consisting of p38 MAPK short hairpin RNA (shRNA) and a control LV were constructed and packaged according to the manufacturer's protocols (GeneChem, Shanghai, China). When the cell confluence was approximately 20%-30%, the p38 MAPK shRNA LV (MOI = 10) or control LV (MOI = 10) was transfected in culture dishes with a HiTransG P infection enhancer (40 *μ*L/mL). Sixteen hours after transfection, the complete medium was replaced, and the culture continued. After 48 hours, the transfection medium was removed, and the cells were incubated with or without BMP4 for 24 hours. Successful endothelial cell transfection was evaluated by quantitative real-time PCR (qRT-PCR) and Western blotting.

### 2.6. Quantitative Real-Time PCR

The p38 MAPK expression level was analyzed via qRT-PCR. Total RNA was extracted from endothelial cells that were sorted from rat mesenteric arteries using TRIzol reagent (Invitrogen, Carlsbad, CA, USA). The RT system protocol for reverse transcription was performed in a 20 *μ*L reaction mixture. Total RNA (1 *μ*g) was used in the reaction, and a random primer was used to initiate cDNA synthesis. The reaction mixture was treated for 10 min at 25°C, 37°C for 120 min, and 85°C for 5 min. SYBR Green PCR core reagents (Applied Biosystems) and an ABI Prism 7500 sequence detection system were used for qRT-PCR. According to the manufacturer's recommendations, qRT-PCR was performed in a 20 *μ*L reaction volume. GAPDH was used as an internal control for p38 MAPK. The 2^-*ΔΔ*CT^ method was used to calculate the relative value compared to the control sample. MAECs were genotyped using the following primers: p38 MAPK-Rat-Forward (GTACCTGGTGACCCATCTCA), p38 MAPK-Rat-Reverse (TCCAATTCAGCATAATCTCG), GAPDH-Rat-Forward (TCAACGGCACAGTCAAGG), GAPDH-Rat-Reverse (ACTCCACGACATACTCAGC).

### 2.7. Western Blot

For Western blot analyses, cell extractions were cleared by 13300 × *g* for 15 min at 4°C. Protein concentration was determined by BCA Protein Assay Kit (APPLYGEN, Beijing, China). The protein samples were separated by 8% SDS-PAGE and transferred to a nitrocellulose membrane using a Trans-blot unit (Bio-Rad Laboratories) for 1.5 hour at 250 mA. The membranes were blocked with 5% (wt/vol) skim milk and 0.1% (vol/vol) Tween-20 in TBS (pH 7.4) for 1 hour at room temperature (25°C). Primary antibodies against BMP4 (1 : 500, ab39973, Abcam, UK), p38 MAPK (1 : 200, sc-7149, Santa Cruz Biotechnology, USA), phospho-p38 (p-p38) MAPK (1 : 1,000, 9216, Cell Signaling Technology, USA), Sgk1 (1 : 500, ab59337, Abcam, UK), p-Sgk1 (1 : 1,000, 44-1260G, ThermoFisher, USA), Nedd4-2 (1 : 500, 4013, Cell Signaling Technology, USA), p-Nedd4-2 (1 : 500, ab168349, Abcam, UK), and GAPDH (1 : 5,000, ab8245, Abcam, UK) were incubated with the membranes overnight at 4°C. After washing with TBS-T, the membranes were incubated for 1 hour at room temperature with the corresponding secondary antibodies (1 : 10,000). All membranes were washed with TBS-T, and the bands were quantified by using the Odyssey infrared imaging system (LI-COR) and Odyssey v3.0 software.

### 2.8. Chemical Reagents

Unless otherwise noted, all chemical reagents used in this study were purchased for Sigma-Aldrich.

### 2.9. Statistics and Data Analysis

All values were expressed as means ± SEM. All data were subjected to the Kolmogorov-Smirnov normality test, and then, parametric or nonparametric analyses were chosen accordingly. If the data were not normally distributed, a nonparametric test (Mann–Whitney test to compare two groups or Kruskal-Wallis test with Dunn's post hoc test to compare three groups) was performed. If the data were found to follow a Gaussian distribution, parametric tests were done (Student's two-tailed *t*-test for comparisons between two groups or one-way ANOVA for multiple groups). Data subjected to ANOVA were followed by Bonferroni's post hoc tests only when the *F* value attained *P* < 0.05, and there was no significant inhomogeneity of variances. Differences were considered statistically significant at *P* < 0.05.

## 3. Results

### 3.1. HS Diet Increases Blood Pressure, Impairs Vascular Relaxation, and Stimulates Endothelial ENaC in SS Rats but Not in SD Rats

**Figure 1:**
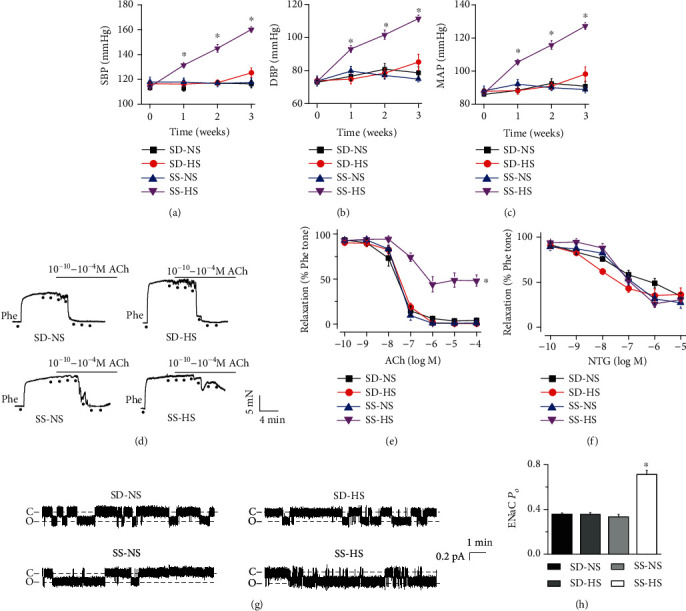
HS diet elevates blood pressure in a time-dependent manner, reduces endothelium-dependent artery relaxation, and stimulates endothelial ENaC in SS rats. SBP (a), DBP (b), and MAP (c) were measured in SD and SS rats fed NS (0.3%) or HS (5%) diet for 0, 7, 14, and 21 days. ^∗^represents *P* < 0.05*vs.* SS-NS, SS rats fed NS (*n* = 7 rats for each group). (d, e) Representative traces of ACh-induced artery relaxation in NS or HS diet-fed SD and SS rats and summary of relaxation responses of mesenteric arteries to different doses of ACh under the conditions shown in (d). The first dot in each plot represents the time when Phe (10 *μ*M) was added. The following dots indicate ACh concentrations gradually increased to 0.1 nM, 1 nM, 10 nM, 100 nM, 1 *μ*M, 10 *μ*M, and 100 *μ*M. ^∗^indicates *P* < 0.05*vs.* SS-NS (*n* = 6 for each group). (f) Summary of relaxation responses of mesenteric arteries from these rats to different doses of NTG (*n* = 6 for each group). (g) Representative ENaC single-channel currents generated by split-open artery technique in intact endothelial cells from NS or HS diet-fed SD and SS rats, under the indicated conditions. (h) Summary of calculated ENaC *P*_*O*_ obtained from single-channel recordings from the different experimental groups as shown in (g). ^∗^represents *P* < 0.05*vs.* SS-NS (*n* = 6 rats for each group).

Our previous studies have shown that the HS diet (8% NaCl) increases systolic blood pressure (SBP) in both SD and SS rats [22, 23]. Here, we found that SS rats fed HS diet (5% NaCl) resulted in a significant increase in SBP, diastolic blood pressure (DBP), and mean arterial pressure (MAP), in a time-dependent manner. However, there were no changes in either SD rats fed the HS diet or NS diet, or in SS rats fed with NS in a period of 3 weeks (Figures 1(a)–1(c)). To determine whether the HS diet altered vascular function in these rats, we used isometric myography to test vascular tone. The data showed that three weeks after feeding the HS diet to SS rats blunted endothelium-dependent artery relaxation (EDR) responses to ACh but had no effect on endothelium-independent artery relaxation responses to NTG. However, EDR was unchanged in HS diet-fed SD rats (Figures 1(d)–1(f)).

To test whether the HS diet enhanced ENaC activity in these rats, we performed cell-attached patch-clamp recordings of ENaC signal-channel currents from intact endothelial cells attached to isolated mesenteric arteries. ENaC activity was significantly higher in HS diet-fed SS rats than in NS diet-fed SS rats; HS diet did not affect ENaC activity in SD rats (Figures 1(g) and 1(h)). These results indicate that the HS diet increases both SBP and DBP, impairs endothelium-dependent vascular relaxation, and stimulates ENaC in SS rats but not in SD rats.

### 3.2. HS Diet Increases BMP4 Expression Both in the Serum and in Endothelial Cells from SS Rats but Not in those from SD Rats

**Figure 2:**
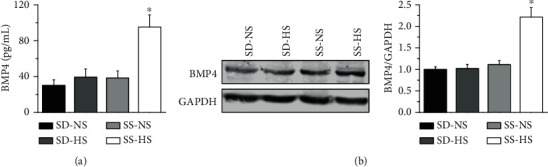
HS diet increases BMP4 levels both in the serum and in the endothelial cells of SS rats. (a) Serum BMP4 levels were measured at day 21 in NS or HS diet-fed SD and SS rats. ^∗^represents *P* < 0.05*vs.* SS-NS (*n* = 13 for each group). (b) Representative Western blot and summarized data showing BMP4 protein levels under each condition in endothelial cells isolated from SD and SS rats fed NS or HS diets. ^∗^represents *P* < 0.05*vs.* SS-NS (*n* = 10 for each group).

We explored whether BMP4 content in HS diet-fed SD and SS rats was increased. The data showed that serum BMP4 levels were increased in HS diet-fed SS rats but not in HS diet-fed SD rats (Figure 2(a)). Moreover, the HS diet significantly enhanced BMP4 expression in endothelial cells from SS rats. In contrast, BMP4 expression in HS diet-fed SD rats showed no significant change (Figure 2(b)). Our results suggest that BMP4 may contribute to high salt-induced hypertension in SS rats.

### 3.3. BMP4 Mediates HS Diet-Induced Impairment of EDR and ENaC Activity in SS Rats

**Figure 3:**
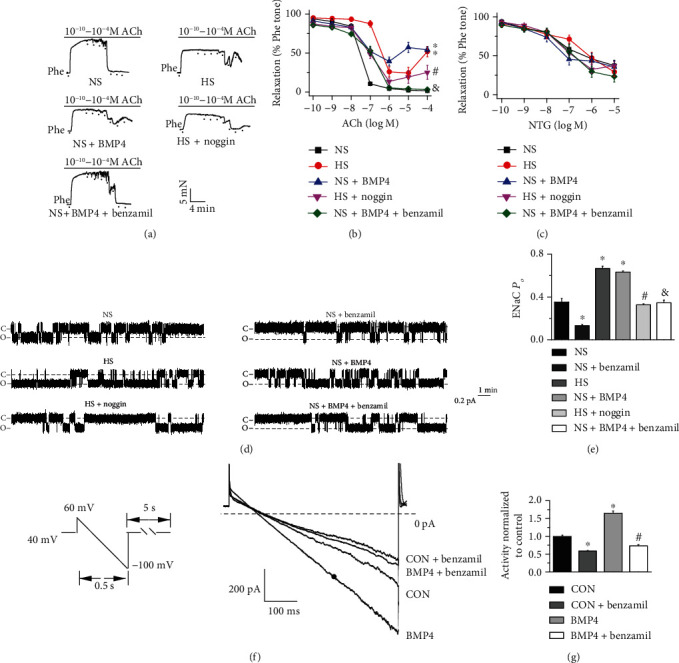
HS diet impairs EDR and stimulates ENaC via a pathway associated with BMP4 in SS rats. (a) Representative traces of ACh-induced artery relaxation in NS or HS diet-fed SS rats; the vascular rings were, respectively, treated with BMP4 (50 ng/mL) and noggin (200 ng/mL) for 4 hours and benzamil (1 *μ*M) for 1 hour before experiments. The first dot in each plot represents the time when Phe (10 *μ*M) was added. The following dots indicate ACh concentrations gradually increased to 0.1 nM, 1 nM, 10 nM, 100 nM, 1 *μ*M, 10 *μ*M, and 100 *μ*M. (b) Summary of relaxation responses of mesenteric arteries to different doses of ACh under the conditions shown in (a). ^∗^indicates *P* < 0.05*vs*. NS; ^#^indicates *P* < 0.05*vs*. HS; ^&^represents *P* < 0.05*vs*. NS + BMP4 (*n* = 6 for each group). (c) Summary of relaxation responses of mesenteric arteries from these rats to different doses of NTG (*n* = 6 for each group). (d) Representative ENaC single-channel currents in intact endothelial cells attached to a mesenteric artery isolated from NS or HS diet-fed SS rats; mesenteric arteries, respectively, treated with BMP4 (50 ng/mL) and noggin (200 ng/mL) for 4 hours and application of 1 *μ*M benzamil prior to recording. (e) Summary of ENaC *P_O_* obtained from different experimental groups as shown in (d). ^∗^*P* < 0.05*vs*. NS, ^#^*P* < 0.05*vs*. HS, ^&^*P* < 0.05*vs*. NS + BMP4 (*n* = 6 rats for each group). (f–g) Representative perforated whole-cell recording setup and summarized data showing the ENaC currents in primary cultured endothelial cells isolated from SS rats. The cells were cultured with or without an additional 20 ng/mL BMP4 in the culture medium for 24 hours, in the presence or absence of 1 *μ*M benzamil. ^∗^indicates *P* < 0.05*vs.* control group; ^#^represents *P* < 0.05*vs.* BMP4 treated group (*n* = 6 individual cells for each group).

Other studies have indicated that BMP4 destroys EDR [15, 27] and that ENaC blockade ameliorates EDR [22]. Therefore, we reasoned that BMP4 may blunt EDR by stimulating ENaC in HS diet-fed SS rats. Incubation of isolated mesenteric artery rings with either a BMP4 antagonist, noggin (200 ng/mL for 4 hours) or with an ENaC blocker, benzamil (1 *μ*M for 1 hour) significantly prevented HS-induced or exogenous BMP4-induced (50 ng/mL treatment for 4 hours) impairment of EDR. In contrast, endothelium-independent relaxation responses to NTG were unaffected (Figures 3(a)–3(c)). These data suggest that BMP4 may lead to endothelial dysfunction by stimulating ENaC in HS diet-fed SS rats.

We then next determined whether HS diet increases ENaC activity through BMP4. We detected a benzamil sensitive ENaC single-channel current in the endothelial cells of split-open SS rat arteries, which the biophysical features including the channel conductance were similar to what we have previously reported [22, 23]. Furthermore, the endothelial ENaC activity was significantly upregulated by HS diet feeding or BMP4 incubation. In contrast, noggin or benzamil treatment abolished the ENaC activity induced by HS diet feeding or BMP4 treatment (Figures 3(d) and 3(e)). Moreover, the data generated from perforated whole-cell patch-clamp recordings showed pretreatment of the primary cultured endothelial cells with BMP4 for 24 hours significantly increased the ENaC currents compared to those of control cells, and the BMP4-induced increase in whole-cell ENaC currents was inhibited by benzamil (1 *μ*M) (Figures 3(f) and 3(g)). These data strongly suggest that the HS diet induces endothelial dysfunction by stimulating endothelial ENaC via BMP4.

### 3.4. HS Diet Results in Endothelial Dysfunction and Increases ENaC Activity through p38 MAPK in SS Rats

**Figure 4:**
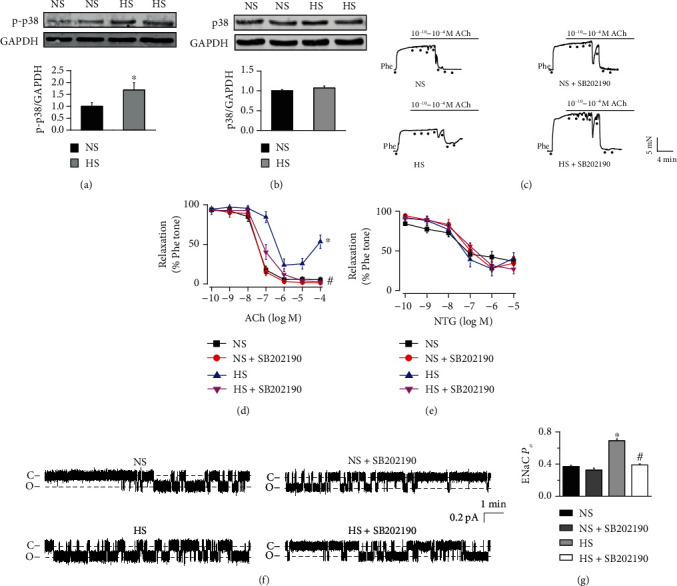
HS diet impairs endothelium-dependent relaxation and stimulates ENaC via p38 MAPK in SS rats. (a, b) Upper panels are representative Western blots and summarized data showing p38 MAPK and p-p38 MAPK protein levels under each condition in endothelial cells isolated from SS rats fed NS or HS diet. ^∗^*P* < 0.05*vs*. NS (*n* = 6 for each group). (c) Representative traces of ACh-induced artery relaxation in NS or HS diet-fed SS rats, in absence of or in the presence of 20 *μ*M SB202190 for 4 hours. The first dot in each plot represents the time when Phe (10 *μ*M) was added. The following dots indicate ACh concentrations gradually increased to 0.1 nM, 1 nM, 10 nM, 100 nM, 1 *μ*M, 10 *μ*M, and 100 *μ*M. (d, e) Summaries of relaxation responses of mesenteric arteries to different doses of ACh (d) and to different doses of NTG (e). ^∗^*P* < 0.05*vs*. NS; ^#^*P* < 0.05*vs*. HS (*n* = 6 for each group). (f) Representative ENaC single-channel currents generated from the intact endothelial cells of mesenteric artery isolated from NS or HS diet-fed SS rats, in the absence of or in the presence of 20 *μ*M SB202190 for 4 hours. (g) Summary of ENaC *P_O_* obtained from single-channel recordings from the SS rats, under indicated conditions, as shown in (f). ^∗^indicates *P* < 0.05*vs.* NS; ^#^represents *P* < 0.05*vs.* HS (*n* = 6 rats for each group).

According to previous studies, p38 MAPK is involved in the pathogenesis of hypertension [28]. To determine whether p38 MAPK mediates HS diet-induced hypertension, endothelial cells were freshly isolated from the mesenteric arteries of HS or NS diet-fed SS rats and subjected to Western blotting. Our data showed that the expression levels of p-p38 MAPK but not those of p38 MAPK were significantly elevated in endothelial cells from HS diet-fed SS rats, but not in those from NS diet-fed SS rats (Figures 4(a) and 4(b)). Next, we determined whether p38 MAPK is involved in HS-induced impairment of EDR. The results showed that HS diet-induced impairment of EDR was reversed by treating the vascular rings with a p38 MAPK inhibitor, SB202190 (20 *μ*M) for 4 hours in SS rats (Figures 4(c)–4(e)). Furthermore, HS-induced increase in endothelial ENaC activity was abrogated by treating the arteries with 20 *μ*M SB202190 for 4 hours prior to performing cell-attached recordings (Figures 4(f) and 4(g)). These results suggest that the HS diet stimulates ENaC and causes endothelial dysfunction in SS rats via p38 MAPK.

### 3.5. HS-Induced Increase in BMP4 Stimulates Sgk1/Nedd4-2 via p38 MAPK

**Figure 5:**
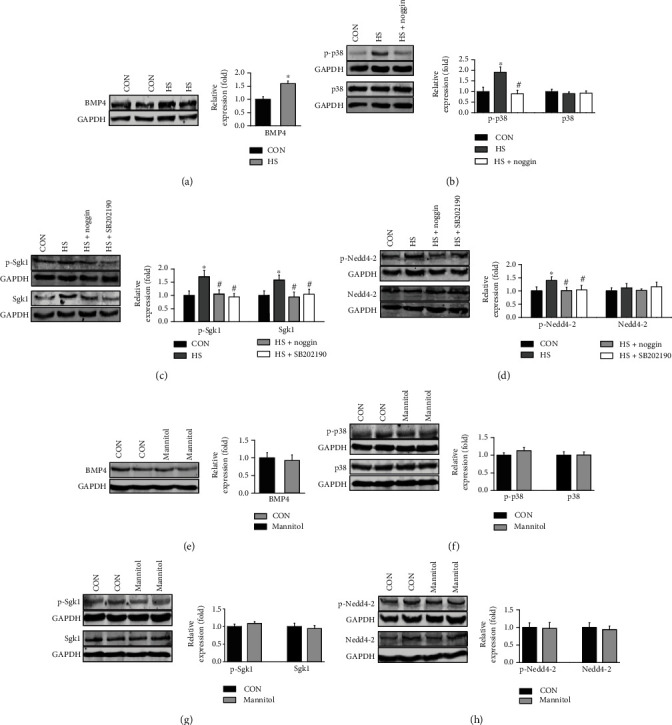
HS activates Sgk1/Nedd4-2 via BMP4 and p38 MAPK signaling. (a) Representative Western blots and summarized data showing BMP4 protein levels in endothelial cells isolated from SS rats either under control conditions or treated with an additional 40 mM NaCl in the culture medium for 24 hours. The data are the means ± SEMs of 6 experiments in each group. ^∗^indicates *P* < 0.05*vs.* control. (b) Representative Western blots and summarized data showing p38 MAPK and p-p38 MAPK protein levels under control conditions or treated with HS additional 40 mM NaCl in the culture medium for 24 hours, in the absence of and in the presence of 100 ng/mL noggin. ^∗^indicates *P* < 0.05*vs.* control; ^#^represents *P* < 0.05*vs.* HS (*n* = 6 for each group). (c–d) Representative Western blots and summarized data showing Sgk1, p-Sgk1, Nedd4-2, and p-Nedd4-2 protein levels under control conditions or treated with additional 40 mM NaCl in the culture medium for 24 hours, in the absence of and in the presence of either 100 ng/mL noggin or 10 *μ*M SB202190 for 24 hours. ^∗^indicates *P* < 0.05*vs.* control; ^#^represents *P* < 0.05*vs.* HS (*n* = 6 for each group). (e–h) Representative Western blots and summarized data showing BMP4, p-p38 MAPK, p38 MAPK, p-Sgk1, Sgk1, p-Nedd4-2, and Nedd4-2 protein levels in primary cultured endothelial cells isolated from SS rats either under control conditions or treated with an additional 80 mM mannitol in the culture medium for 24 hours. The data are the means ± SEMs of 6 experiments in each group.

Since p-p38 MAPK in hypertensive rats is enhanced by BMP4 [28], we then examined whether BMP4 may stimulate the levels of active (phosphorylated) Sgk1/Nedd4-2 via p38 MAPK to elevate ENaC activity by reducing ENaC ubiquitination. To reduce the number of animals used, endothelial cells were freshly isolated from the mesenteric arteries of SS rats fed NS diet, amplified in culture, and treated with NaCl (additional 40 mM in culture media), NaCl (additional 40 mM in culture media) + noggin (100 ng/mL), or NaCl (additional 40 mM in culture media) + SB202190 (10 *μ*M) for 24 hours. Our data showed that BMP4 and p-p38 MAPK were significantly elevated by additional NaCl in the culture medium and that the increases in p-p38 MAPK were reversed by noggin (Figures 5(a)–5(b)). It has been revealed that enhanced phosphorylation levels of Sgk1/Nedd4-2 prevents Nedd4-2 mediated ENaC degradation and increases ENaC activity [29–31]. Therefore, we next explored whether p38 MAPK and its upstream signal BMP4 could increase the levels of phosphorylated Sgk1/Nedd4-2 in these endothelial cells. The data showed that total Sgk1, p-Sgk1, and p-Nedd4-2 were significantly increased in HS-treated primary cultured endothelial cells and that the increases were abolished by noggin or SB202190 (Figures 5(c)–5(d)). We then examined whether the effect of high salt on the expression of these proteins in endothelial cells was due to the change in osmolarity. As the osmolarity of 80 mM of mannitol equals the osmolarity of 40 mM of NaCl [32], 80 mM mannitol used to examine whether osmolarity can alter the expression levels of these proteins. The data showed that mannitol at a concentration of 80 mM did not affect the expression levels of BMP4, p-p38 MAPK, p38 MAPK, p-Sgk1, Sgk1, p-Nedd4-2, and Nedd4-2 (Figures 5(e)–5(h)), suggesting the effect of high salt on the expression levels of these proteins were not due to changes in osmolarity. These data suggest that HS stimulates Sgk1/Nedd4-2 signaling through BMP4 and p38 MAPK.

### 3.6. p38 MAPK Mediates BMP4-Induced ENaC Activity by Stimulating Sgk1/Nedd4-2

**Figure 6:**
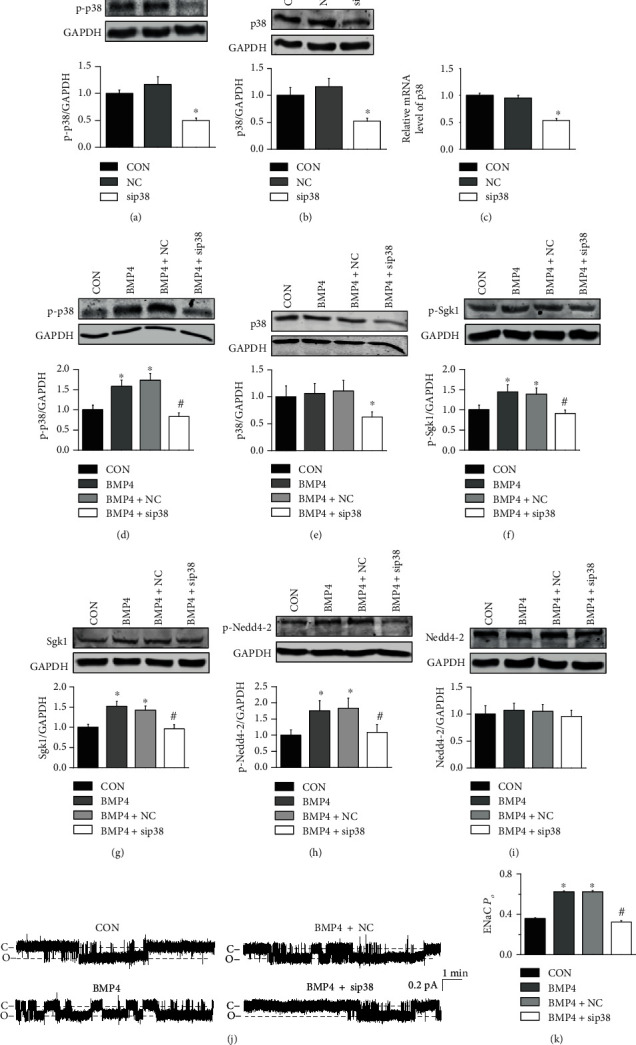
BMP4 stimulates ENaC by activating p38 MAPK and Sgk1/Nedd4-2. The experiments were conducted in the primary cultured endothelial cells (ECs) isolated from SS rats and the ECs were, respectively, transfected by p38 MAPK control LV (MOI = 10) and p38 MAPK shRNA LV (MOI = 10) to knockdown p38 MAPK. (a, b) Representative Western blots and summarized data showing p38 MAPK and p-p38 MAPK protein levels in primary cultured endothelial cells isolated from SS rats either under control conditions or treated with p38 MAPK control LV or p38 MAPK shRNA LV. ^∗^*P* < 0.05*vs.* control (*n* = 6 for each group). (c) Summarized data showing p38 MAPK mRNA levels in primary cultured endothelial cells isolated from SS rats either under control conditions or treated with p38 MAPK control LV or p38 MAPK shRNA LV. ^∗^*P* < 0.05*vs.* control (*n* = 6 for each group). (d–i) Representative Western blots and summarized data showing p38 MAPK, p-p38 MAPK, Sgk1, p-Sgk1, Nedd4-2, and p-Nedd4-2 protein levels in ECs or in p38 MAPK gene manipulated ECs under control conditions or treated with 20 ng/mL BMP4 for 24 hours. ^∗^indicates *P* < 0.05*vs.* control; ^#^represents *P* < 0.05*vs.* BMP4 (*n* = 6 for each group). (j) Representative ENaC single-channel currents recorded from ECs or in p38 MAPK gene manipulated ECs, in the absence of or in the presence of 20 ng/mL BMP4 (24 hours). (k) Summary of ENaC *P_O_* obtained from the experiments shown in (j). ^∗^indicates *P* < 0.05*vs.* control; ^#^represents *P* < 0.05*vs.* BMP4 (*n* = 6 for each individual cells).

To confirm the role of p38 MAPK in mediating BMP4-induced ENaC activity, we knocked down p38 MAPK gene expression using a shRNA LV. We found that protein expression levels of p38 MAPK and p-p38 MAPK, p38 MAPK mRNA levels were reduced, whereas the negative control (NC) LV did not affect p38 MAPK or p-p38 MAPK (Figures 6(a)–6(c)). Moreover, Western blot data revealed that the expression levels of p-p38 MAPK were significantly increased by BMP4, and the effects of BMP4 on p-p38 MAPK were abolished by p38 MAPK shRNA transfection (Figures 6(d) and 6(e)). Furthermore, BMP4-induced in an increase in p-Sgk1, Sgk1, and p-Nedd4-2 levels were reversed p38 MAPK shRNA infection, whereas BMP4 did not affect Nedd4-2 expression (Figures 6(f)–6(i)). These results suggest that BMP4 stimulates Sgk1/Nedd4-2 through p38 MAPK. Furthermore, the data showed that knocking down p38 MAPK attenuated BMP4-induced increase in ENaC activity in the primary cultured endothelial cells isolated from SS rats (Figures 6(j) and 6(k)). These data together indicate that BMP4 increases ENaC activity by stimulating Sgk1/Nedd4-2 via p38 MAPK.

## 4. Discussion

In the present study, for the first time, we show that high salt intake induces overexpression of BMP4 in the serum and endothelial cells of salt-sensitive rats, that BMP4 stimulates ENaC in endothelial cells via a pathway associated with p38 MAPK and Sgk1/Nedd4-2, and that BMP4 decreases EDR by stimulating ENaC. These results may have clinical significance for managing hypertension in salt-sensitive populations and for improving the treatment of colorectal adenocarcinoma patients with hypertension.

We have previously shown that the HS diet (8% NaCl) increases SBP in both SD and SS rats [22, 23]. Since a diet containing approximately 0.3% NaCl is considered an NS diet [33], the HS diet we previously used 8% NaCl contains almost 30 times more salt than the NS diet. This substantial difference could be the reason that the HS diet increases BP in both SD and SS rats. Therefore, we used a reduced HS diet (5% NaCl) for the present study. According to a simple practice guide for dose conversion between rat and human [34], when rats feeding a HS diet with 5% NaCl content, it is equivalent to a person weighing 60 kg eating about 46.2 g of NaCl per day. The HS diet used in SS hypertension is much higher than the salt in human diet. However, such high concentrations have been extensively used to produce a SS hypertension model in SS rats [35–37]. Indeed, the data showed that 5% NaCl increases BP and BMP4 only in SS rats but not in SD rats.

We demonstrate that in SS rats BMP4 plays a crucial role in HS-induced ENaC activity and endothelial dysfunction via p38 MAPK-dependent activation of Sgk1/Nedd4-2. In contrast to TGF-*β* which causes endothelial dysfunction in SD rats in response to a relatively high salt (8%) diet [38], our data suggested that BMP4 may be a signaling molecule that mediates HS-induced hypertension specifically in SS populations. Indeed, BMP4 infusion by osmotic pumps increased systolic blood pressure in a time- and dose-dependent manner in both C57BL/6 mice and apolipoprotein E-null mice [15]. We favour the notion that increased BMP4 is a reason for rather than a result of hypertension, because the administration of BMP4 causes hypertension [15].

In the present study, we did not show the mechanism by which HS diet increases serum BMP4. Since our data showed that the HS diet also increases BMP4 in endothelial cells, we argue that increased serum BMP4 in response to HS challenge may come from endothelial cells as a paracrine signaling molecule. This argument is also supported by a previous study showing that BMP4 is expressed in endothelial cells and can induce endothelial dysfunction [39]. However, increased serum BMP4 may also come from other tissues, because HS intake stimulates the expression of BMP4 in renal cortex tissue [40]. It remains unclear how HS promotes BMP4 expression. Previous studies have shown that aldosterone promotes BMP4 expression in the kidney [40]. Interestingly, we showed that the HS diet elevates plasma aldosterone in SS rats [22]. These studies together suggest that HS may elevate serum BMP4 by elevating plasma aldosterone. However, inconsistent results also exist, showing that aldosterone decreases BMP4 levels in mouse mesangial cells [41]. Nevertheless, the present study suggests that BMP4 mediates HS-induced endothelial dysfunction and hypertension. Since previous studies have shown that BMP4 infusion induces hypertension in mice in a vascular NADPH oxidase-dependent manner and the subsequent endothelial dysfunction [15], BMP4 may act as a paracrine signaling molecule to modulate endothelium-dependent vascular relaxation in salt-sensitive hypertension.

The present study is the first evidence showing that BMP4 stimulates ENaC. We also showed that the downstream signaling transduction is associated with phosphorylation of p38 MAPK and then Sgk1/Nedd4-2. This pathway is not surprising, because it is known that p-p38 MAPK is elevated in hypertensive rats fed HS/high-fat diet [42], and phosphorylation of Nedd4-2 by Sgk1 increases ENaC activity by reducing its degradation [29–31]. It is also known that BMP4 activates p38 MAPK in mouse endothelial cells via a ROS-dependent mechanism and that BMP4 causes endothelial dysfunction that can be reversed by a p38 MAPK inhibitor [25]. Furthermore, a p38 MAPK inhibitor can prevent HS-induced Sgk1 expression in inner medullary collecting duct cells [43]. However, inconsistent results also exist, showing that p38 MAPK reduces ENaC activity in mice [44]. We first showed that ENaC can be stimulated by the Sgk1/Nedd4-2 in endothelial cells. Since a p38 MAPK shRNA lentivirus could prevent BMP4-induced Sgk1, p-Sgk1, and p-Nedd4-2 activation, p38 MAPK should be the immediate signaling molecule after BMP4 but before Sgk1 and Nedd4-2 in the pathway. We argue that increased ENaC activity by BMP4 in response to HS diet may account for reduced vascular relaxation, because the data show that the relaxation can be abolished by benzamil, an ENaC blocker. This argument is also supported by our previous studies showing that increased ENaC activity impairs vascular relaxation in nitric oxide (NO)-dependent manner [22]. We have also shown that blockade of ENaC prevents ox-LDL-induced vascular dysfunction by increasing NO [24]. It has been shown in Liddle mice that activated ENaC is responsible for reduced NO production and contributed to vascular endothelial cell stiffness [45]. How ENaC in endothelial cells works with ENaC in the distal nephron to mediate the pathogenesis of SS hypertension must still be determined.

## 5. Conclusion

HS intake elevates BMP4 in endothelial cells and serum of SS rats. The elevated BMP4 stimulates ENaC in endothelial cells via a pathway associated with p38 MAPK and Sgk1/Nedd4-2. BMP4 induced by HS decreases endothelium-dependent artery relaxation by stimulating ENaC. These results may have clinical significance for the management of hypertension in SS populations.

## Data Availability

The data used to support the findings of this study are available from the corresponding author upon request.

## Abbreviations

BMP4: Bone morphogenetic protein 4
ENaC: Epithelial sodium channel
SD: Sprague-Dawley
SS: Salt-sensitive
NS: Normal salt
HS: High salt
EDR: Endothelium-dependent relaxation
p38 MAPK: p38 mitogen-activated protein kinase
p-p38: Phospho-p38
Sgk1: Serum/glucocorticoid regulated kinase 1
Nedd4-2: Neural precursor cell-expressed developmentally downregulated protein 4-2
MAEC: Mesenteric artery endothelial cell
ECs: Endothelial cells
TGF-*β*: Transforming growth factor-*β*
PSS: Physiological saline solution
ACh: Acetylcholine
Phe: Phenylephrine
NTG: Nitroglycerin
LV: Lentivirus
shRNA: Short hairpin RNA
qRT-PCR: Quantitative real-time PCR
SBP: Systolic blood pressure
DBP: Diastolic blood pressure
MAP: Mean arterial pressure
NC: Negative control
NO: Nitric oxide
*P_O_*: Open probability.
